# Integration between the knowledge diaspora and their country of origin through institutional affiliations: a case study for policy development

**DOI:** 10.3389/frma.2026.1852906

**Published:** 2026-07-03

**Authors:** Horacio G. Rotstein

**Affiliations:** Federated Department of Biological Sciences, New Jersey Institute of Technology and Rutgers University, Newark, NJ, United States

**Keywords:** brain drain, brain integration, CONICET-Argentina, corresponding researchers, diaspora networks, honorary affiliations, science diplomacy, scientific diaspora

## Abstract

The knowledge economy is fundamentally driven by human capital, making the “brain drain” phenomenon a critical challenge for national development. Traditionally, policy frameworks have focused on repatriation, circulation or vinculation to address this issue. However, these models often fail to account for more regular and permanent institutional associations between the diaspora of knowledge professionals (KPs) and the science, technology and innovation (STI) institutions of their country of origin (CO). Such associations are increasingly feasibly due to technological advances that render many STI activities independent of geography. This paper advances the notion of KP diaspora *integration* within the domestic STI institutions of their CO, incorporating a framework focused on structural institutional membership. We provide an in depth analysis and propose a blueprint for policy development aimed at achieving this goal. We use Argentina as a case study for several reasons, including its well-developed STI system, its comparatively large KP diaspora, and its history of KP diaspora organization and interaction with the Argentine STI system. The proposed policy framework opens avenues for the development of specific policies at the institutional level tailored to the unique characteristics, local constraints and goals of each organization. The ideas, core principles, and policy blueprint we present have a broader generality and are applicable *mutatis mutandis* to other developing countries experiencing the brain drain phenomenon.

## 1 Introduction

Knowledge has rapidly become the primary driving force for economic development (Organisation for Economic Co-operation and Development, 1996; David and Foray, 2002; Mangabeira Unger, 2019; Drucker, 1993). Because knowledge is inherently a human activity, *knowledge economies*—those based on the creation, dissemination and use of knowledge and information (Organisation for Economic Co-operation and Development, 1996)—are increasingly dependent on their human capital (Romer, 1990; Becker, 1964; Schultz, 1961)—broadly defined the collective skills, knowledge, experience and health of a population—which determines their competitive advantage. Science, technology and innovation (STI) are central to the notion of intellectual capital and have emerged as fundamental engines of progress. This is driven by a broad spectrum of *knowledge professionals* (KPs)—those whose primary contribution to the economy and society is the creation, application and distribution of theoretical and practical knowledge, and the management of information and specialized expertise—working in academia, industry and other organizations. The training of the next generation of KPs ensures that the intellectual capital is renewed and expanded, and therefore secures the STI pipeline.

The *brain drain phenomenon*—the considerable emigration of KPs from one country (country of origin; CO) to another (country of residence, typically more developed)—carries significant negative consequences for the economy of the CO (Adams, 1964; Boeri et al., 2012; Bonilla, 2024a; Docquier et al., 2007; Emólieva, 2010; Greenberg, 1964; McVittie, 1967; Weinberg, 2011; Ozden, 2005; Dodani and LaPorte, 2005; Portes, 1976; Watanabe, 1969). Knowledge economies suffer not only the immediate loss of active KPs and the resulting decline in STI productivity, but also the forfeited return on investment in their training and education and the loss of a significant fraction of the next generation of trained KPs.

Several strategies have emerged to address these challenges and alleviate—even try to reverse—the impact of brain drain (García de Fanelli, 2008; Hunter, 2013; Zweig, 2006; Heitor et al., 2014), including *repatriation* (Carranza, 2015; Vera and López, 2023; Bayle, 2015), *circulation* (Saxenian, 2002; Johnson and Regets, 1998; Shin and Moon, 2018; Tung, 2008), *vinculation* (Leiva, 2005; Bastías, 2023; Granés et al., 1996; Chaparro et al., 2004; Kramer and Zent, 2019), and *exchange* (García de Fanelli, 2008; Emólieva, 2011; Pellegrino, 2008) (see the definitions in Section 7 of Supplementary material). Repatriation is often a practically unfeasible and unrealistic expectation beyond certain career stage. The remaining strategies reflect the evolving perspective on the KP diaspora along the *brain drain*—*brain gain* axis (Shin and Moon, 2018; Agrawal et al., 2011; Aupetit, 2009; Dodani and LaPorte, 2005; Hunter, 2013; Thomas and Mathew, 2020; Batista et al., 2025), which has undergone a dramatic paradigm shift (Echeverría-King et al., 2022; Bonilla et al., 2023; Caloz-Tschopp, 2010; Meyer, 2001; Tejada, 2012; Meyer and Brown, 1999; Kramer and Zent, 2019; Pombo et al., 2025; Piñeros Ayala et al., 2025; Coelho Ferreira et al., 2025; Hernández Mondragón et al., 2025; Burns, 2013; Shin and Moon, 2018; Shin and Caywood, 2025). Contemporary frameworks have moved beyond the binary view of human capital gain-loss to emphasize the importance of engaging with KP diaspora networks (Epstein and Heizler, 2016; Chaparro et al., 2004; Pombo et al., 2025; Kuznetsov, 2006; Shin and Caywood, 2025; Meyer and Wattiaux, 2006; Agrawal, 2017; Meyer, 2007) as essential vehicles of linkage with the global STI landscape. However, vinculation and related strategies, though valuable, tend to be occasional, project-specific, and temporary, lacking the long-term commitment required for sustainable impact. Moreover, within the existing frameworks, the KP diaspora remains largely external to the STI institutions of their CO, and the interactions between KP diaspora members and their domestic counterparts remain restricted to individual initiatives rather than being supported by formal institutional structures.

In view of this, we have recently advanced the notion of *integration* (Rotstein and Galvis, 2023), metaphorically framing the KP diaspora as “members of the tribe”. This framework moves beyond vinculation by incorporating a model of structural membership that allows diaspora KPs to be affiliated with domestic STI institutions by courtesy appointment mechanisms. This is facilitated by the continuous advances in communication and the increasing ability to interact remotely. The envisioned tight institutional relationship between domestic and overseas KPs aims to foster robust international collaborations and generate a return in the development of the next generation of domestic KPs. The success of such an integration policy requires the design and implementation of institutional mechanisms that promote and facilitate the interaction between domestic and diaspora KPs and foster a sense of institutional belonging. These mechanisms must be flexible and institutionally specific, allowing for continuous monitoring and refinement, and informed by the lived experiences of the actores involved. While there has been extensive policy-making activity regarding vinculation, a conceptual policy framework regarding integration is lacking and the design of robust integration institutional policies is remarkably rare.

This paper along with the proposed policy framework addresses this critical gap. Drawing on Argentina as a case study, we developed a blueprint for policy recommendations aimed to institutionally integrate the KP diaspora within the domestic STI system. Argentina is a paradigmatic case. It has a well-developed STI system primarily centered at the National Council of Scientific and Technological Research (CONICET) and a well-developed network of public research universities (Bayle, 2015; Pombo et al., 2025; Kreimer, 1997). At the same time, it has the largest KP diaspora of all Latin American countries (Jonkers and Cruz-Castro, 2013; Meyer, 2001). There is a long tradition of scientific collaborations between members of the diaspora and their colleagues in Argentina. Members of the diaspora have organized into associations and networks over the last several decades, catalyzed either by diaspora-led initiatives or by official state-led efforts (Leiva, 2005; Lértora Mendoza, 1996; Pellegrino, 2002; Carranza, 2015; García de Fanelli, 2008; Bayle, 2015; Pombo et al., 2025). In recent years, Argentina has developed an official engagement program with the KP diaspora as part of the RAICES program (Gómez Hernández, 2021; Pombo et al., 2025; Law, 2008). Networks of Argentine scientists abroad exist in more than twenty countries.^1^ While institutionally autonomous, they are affiliated with the RAICES program and regularly interact with the STI institutions in Argentina. While, arguably, CONICET has been a pioneer in the notion of integration (CONICET, 1987), most of the Argentine efforts have been focused on repatriation and vinculation, leaving a policy-vacuum regarding integration. However, the factors mentioned above render the groundwork fertile for addressing this gap.

While the analysis and framework refer specifically to the Argentine STI system and its KP diaspora, the ideas, core principles and policy blueprint possess a broader generality and they apply *mutatis mutandis* to other developing countries facing similar challenges.

A list of Acronyms is provided in Table 1. Precise definitions of several key concepts are presented in Section 7 of the Supplementary material.

**Table 1:** List of acronyms.

CONICET	National Council of Scientific and Technological Research (*Consejo Nacional de Investigaciones Científicas y Técnicas*) Argentina
CR	Corresponding Researcher (CONICET) (*IC; Investigador Correspondiente*)
CO	Country of origin
EP	Extraordinary Professor or equivalent at universities (*Profesor Extraordinario*)
KP	Knowledge Professional
MinCyT	Ministry of Science, Technology and Innovation, Argentina (*Ministerio de Ciencia, Tecnología e Innovación*)
RCAE	Network of Argentine Scientists Abroad (*Redes de Científicos Argentinos en el Exterior*)
RAICES	Red de Argentinos Investigadores Científicos en el Exterior Raíces is also Spanish for roots
SD	Science Diplomacy
STID	Science, Technology & Innovation Diplomacy
STI	Science, Technology and Innovation
ST	Science & Technology
SeICT	Secretariat of Innovation, Science and Technology, Argentina (*Secretaría de Innovación, Ciencia y Tecnología*) (*Jefatura de Gabinete de Ministros de la República Argentina*)

## 2 Background, context, diagnosis, and vision

### 2.1 Brain drain and brain gain

In recent decades, the *brain drain* phenomenon (Adams, 1964; Balmer et al., 2009; Boeri et al., 2012; Bonilla, 2024a; Docquier et al., 2007; Emólieva, 2010; Greenberg, 1964; McVittie, 1967; Meyer and Brown, 1999; Tigau, 2010, 2013; Weinberg, 2011; Portes, 1976; Ozden, 2005; Dodani and LaPorte, 2005; Carrington and Detragiache, 1999) has affected Argentina (Albornoz et al., 2002; Aupetit and Gérard, 2009; Babini et al., 1992; Bonilla, 2024b; Calvelo, 2011; Horowitz, 1962; Houssay, 1966; Lattes et al., 1986; Leiva, 2005; Lértora Mendoza, 1996; Luchilo, 2011; McKee, 1985; Pellegrino, 2002; Oteiza, 1965; Solimano, 2003; Trotman, 2022; Rivero and Navarro-Conticello, 2021; Bayle, 2015; Pombo et al., 2025), a country with a long history of scientific and technological (ST) development and a strong tradition in human resource training (Albornoz and Gordon, 2011; Bekerman, 2016; Buchbinder, 2005; Del Bello and Barsky, 2021; Myers, 1992; Vasen, 2012, 2013; Vessuri, 2007). Scientists, researchers, and, more generally, knowledge professionals (KPs) have emigrated for multiple and complex reasons, including personal, political, economic, academic, and institutional instability. Several mitigating strategies have been proposed and implemented around the *brain drain—brain gain* axis (Carranza, 2015; García de Fanelli, 2008; Pellegrino, 2008; Hunter, 2013; Zweig, 2006; Heitor et al., 2014) such as *circulation* (Saxenian, 2002; Johnson and Regets, 1998; Shin and Moon, 2018; Tung, 2008), *exchange* (García de Fanelli, 2008; Emólieva, 2011; Pellegrino, 2008), *vinculation*^2^ (Leiva, 2005; Bastías, 2023; Granés et al., 1996; Chaparro et al., 2004; Kramer and Zent, 2019) (but see (Chaparro et al., 2006)), and *repatriation* (Carranza, 2015; Vera and López, 2023; Bayle, 2015) with varying degrees of success.

### 2.2 KP diaspora integration

We define KP diaspora integration as the formal affiliation of members of the KP diaspora with an STI institution, broadly understood, in the CO through courtesy appointments or similar mechanisms. A courtesy appointment is a non-permanent, unpaid position granted to someone already employed in another institution, recognizing their contributions (e.g., guest lecturing, advising, research collaboration) without forming an employer-employee relationship and without granting voting rights. In tenure systems, these positions are non-tenure-track. Members of the KP diaspora maintain their regular primary affiliations with their home institutions in their country of residence. For the reminder of this paper we will use KP diaspora integration and under the assumption that the context of “within the STI institutions of their CO” is implied and does not need to be explicitly stated.

The advancement of this notion of *integration* (Rotstein and Galvis, 2023) is driven by the recognition of various factors. Vinculation and exchange have yielded significant and positive results, but tend to be sporadic, occasional, and focused on a specific purpose. Integration, on the other hand, has a regular and sustained nature that aims to consider the KP diaspora as “members of the house” and not merely visitors or hosts. Integration goes beyond vinculation, exchange, *brain sharing* (Thomas and Mathew, 2020) or international *brain mobility* (Aupetit and Gérard, 2009; Aupetit, 2009) in that is not limited to sharing knowledge and expertise between colleagues in the diaspora and their CO (Trotman, 2022), but it involves an institutional affiliation. This type of synergistic interactions is at the heart of the role that KP diasporas and the networks to which they belong play in science diplomacy (SD) (Society, 2010; Flink and Schreiterer, 2010; Gluckman et al., 2017; Ruffini, 2017; Burns, 2013; Wren, 2014; Castells, 1996; Kuznetsov, 2006; Meyer and Brown, 1999; Barabási, 2002; Barabási and Pósfai, 2016).

Repatriation of KPs (Alfaro and Chávez Elorza, 2020; Rivero and Trejo-Peña, 2020), while desirable and successful in some cases, is generally limited and unfeasible—especially for KPs who are already established and have “put down roots” in their countries of residence (CONICET, 1987; Bayle, 2015)^3^. Moreover, repatriation of established KPs is costly and does not clearly present itself as a good investment upfront. This is in contrast to the return of early-career KPs who have completed their training or specialization abroad and are on their path to independence.^4^ While one could argue whether this constitutes repatriation or not (e.g., circulation), it appears to be sensible to focus efforts on supporting the return of this population. Finally, advancements in communication technologies significantly facilitate interpersonal interactions, making them virtually independent of geography, with the consequently positive effects on the development of scientific collaborations and human resource training across borders.

### 2.3 KP diaspora integration: a hypothesis

Our underlying hypothesis is that the tight integration of the KP diaspora—while maintaining their primary ties with institutions in their country of residence—directly contributes to the development of the knowledge economy in the involved countries and fosters international collaborations (David and Foray, 2002; Organización Internacional para las Migraciones (Autor Institucional), 2007). Integration transcends the network society proposition (Castells, 1996), which primarily views the KP diaspora as static nodes in a highly interconnected network that links their CO to global innovation hubs. Instead, the integration framework redefines these networks to have a dual and dynamic topology where the KP diaspora nodes are tightly linked to hubs within the CO's cluster, while remaining situated within external global hubs.

### 2.4 KP diaspora integration: presence in CONICET early policy

Although references to integration are almost inexistent in the literature, with most discussions focusing on vinculation, exchange and repatriation, the idea of integration has not been absent from Argentina's ST policy. In fact, the notion of integration can be traced back to CONICET's 1987 regulations regarding the establishment of the figure of *Corresponding Researcher* (CR) (CONICET, 1987) and persists in subsequent policies (CONICET, 2006, 2015, 2022b,a) (see details in Sections 1 and 2 in Supplementary material).

The notion of integration is also embedded in the so-called RAICES Law (Law, 2008), a state policy which established the RAICES program (Bastías, 2023; Trotman, 2022; Gómez Hernández, 2021; Vera and López, 2023; Ugartemendía, 2021; Pombo et al., 2025). While international collaborations between Argentine KPs abroad and in Argentina are common and have existed over the last many decades, this primarily falls into the category of vinculation.

### 2.5 KP diaspora: interactions with the Argentine STI system and the RAICES program

The notion of integration is informed by the extensive, rich, and productive experience of the dynamic relationship between the diaspora of KPs and the Argentine STI system at individual, group, and institutional levels (CONICET, 1987, 2006, 2015). This is evidenced by the more than one hundred RAICES Awards (Pombo et al., 2025) ^5^ granted since 2010 to Argentine KPs residing abroad in recognition of their collaborative activities with the Argentine STI system, as well as the numerous joint publications between Argentine scientists abroad and in Argentina. Indeed, over the last decades, Argentine researchers abroad have collaborated with colleagues in Argentina on research projects, co-authored publications, contributed to human resource training both in Argentina and at their institutions abroad, participated in project evaluations, served as jurors in academic and administrative competitions at Argentine institutions, taught courses at Argentine universities, and participated in the creation of KP diaspora networks, among other activities. These efforts have stemmed from a combination of individual initiatives and institutional programs from universities, research institutes, and MinCyT (now SeICT), primarily through the RAICES program, facilitated by tools such as the above-mentioned Milstein Fellowship^6^ and other support programs within the university system.

### 2.6 KP diaspora: the networks of Argentine scientists abroad

The networks (Aupetit and Gérard, 2009; Carranza, 2015; García de Fanelli, 2008; Kuznetsov, 2006; Meyer and Brown, 1999; Meyer, 2001; Pedrosa, 2011; Renaud, 2009; Tejada, 2012; Tigau, 2010) that bring together members of the diaspora of KPs play a fundamental role in any architecture of both vinculation and integration ^7^ (Obiol, 2016). Over the last decades, various organizations of Argentine KPs abroad^8^ have been established either by diaspora-led initiatives or official efforts (Leiva, 2005; Lértora Mendoza, 1996; Pellegrino, 2002; Carranza, 2015; García de Fanelli, 2008; Bayle, 2015; Pombo et al., 2025). The currently active *Networks of Argentine Scientists Abroad* (*Redes de Cient*í*ficos Argentinos en el Exterior*; RCAE)^9^, affiliated with the RAICES Program in the framework of the Raíces Law (Law, 2008), are part of this tradition and have contributed through various integration initiatives with the Argentine STI system for more than a decade. These networks and their members are natural actors of SD and agents of development [(Echeverría-King et al., 2022; Ruffni, 2020; Ruffini, 2017; Society, 2010; Flink and Schreiterer, 2010; Gluckman et al., 2017; Turekian, 2018; Rungius et al., 2018; S4D4C, 2019; Colglazier, 2024; Turchetti and Lalli, 2020; Leshner, 2014; Moonwaw, 2018; Lord and Turekian, 2017; Fedoroff, 2009; Copeland, 2016; Rotstein, 2025b; Pérez et al., 2025; Cohen, 1997; Kuznetsov, 2006; Tejada, 2012; Ezekiel and Mauduit, 2023; Tejada, 2012); Organización Internacional para las Migraciones (Autor Institucional), 2007]. The combination of autonomy and affiliation and the experience and knowledge accumulated by these networks collectively as well as individually by their members is of utmost importance for the design and analysis of integration policies such as the ones we propose in this paper. There is a risk that such valuable resources may be underutilized if appropriate mechanisms are not put in place to channel and leverage them effectively.

### 2.7 KP integration: mechanisms

Any integration architecture requires the development of appropriate mechanisms that promote and facilitate the interaction of the KP diaspora with the local STI system, on one hand, and provide the KP diaspora members with a sense of belonging, on the other.

Integration mechanisms and tools should be framed within a comprehensive, flexible and decentralized policy of STI diplomacy (STID) (Colglazier, 2024; Copeland, 2016, 2011; Fedoroff, 2009; Flink and Schreiterer, 2010; Flink and R uffin, 2019; Gluckman et al., 2017; Ruffini, 2017; Society, 2010; Turekian, 2018) that is needed to navigate the complex institutional landscapes and meet the stated goals. Finally, as with any other mechanism in this field, a certain level of investment and evaluation of outcomes—beyond anecdotal evidence—is necessary to ensure effectiveness and long-term impact.

Mechanisms of the type mentioned above are not strange to the university and STI system. In fact, they have existed for decades, but need to be properly adapted and fully leveraged to meet the specific needs of KP integration. These mechanisms include not only the CONICET's CRs mentioned above, but also the category of Extraordinary Professors (EPs) or their equivalents in universities, which appear in the statutes of many—if not most—of them (e.g., see details in Sections 3–6 in Supplementary material). These types of affiliations grant a special institutional status to certain individuals based on their merits, contributions, distinguished services, potential impact, and commitment to the institution. However, they do not entail the usual obligations of regular members nor grant them full rights, which everyone retains with their primary, home institution. While these mechanisms exist, albeit some only in principle, it is unclear whether they align with current realities, needs, and evolving technological possibilities. Furthermore, there is uncertainty as to whether they are sufficiently flexible to accommodate the present requirements and circumstances of the KP diaspora.

### 2.8 Corresponding researchers at CONICET

The figure of CR at CONICET has existed since 1987 (CONICET, 1987, 2006, 2015; Lértora Mendoza, 1996) and remains in effect today. This category is honorary, intended for established researchers, and serves as a complement to the regular categories within the Scientific and Technological Researcher career (see Sections 1 and 2 in the Supplementary material). It is designed for^10^.

“…active Argentine and foreign researchers who do not belong to the Researcher Career, with permanent residence abroad or in Argentina, of recognized prestige, and with outstanding contributions to science and technology. They participate or collaborate in activities such as supervising fellows or researchers, leading or taking part in research projects, teaching courses, and engaging in any activities that contribute to the development of scientific and technological research in Argentina.”

The first modification to the policy occurred in 2006 (CONICET, 2006), followed by a second revision in 2015 (CONICET, 2015), which remains in effect today. the admission process can be initiated either by the interested individual or by the authorities of various institutions and must be approved by the Directorate (see Section 2 in Supplementary material). In 2022, for the first time, a call for applications (CONICET, 2022b,a) was issued based on the 2015 policy. Section 2 in Supplementary material details the requirements, admission process, and commitments associated with the CR status, among other aspects, as well as the historical evolution of these since their inception. In its original version (1987)—but not in subsequent versions (2006, 2015)—this mechanism included the possibility of paid visits by CRs to Argentina and required at least one visit during the 6-year designation period.

The figure of CR at CONICET has not been free from criticism and proposals for improvement. Reports in the literature (Lértora Mendoza, 1996) suggest that the experience in its initial phase was not as successful as expected due to various factors, including the impracticality of certain requirements and evaluation mechanisms. Requests made after the initial period (1988/89) were apparently not addressed in a timely and appropriate manner. (It is important to bear in mind that international communication resources were significantly more precarious than they are today. It is unknown to us whether the situation changed since the publication of the previous reference.) Some of these issues were tackled in subsequent modifications. As of the time of writing these notes, there appears to be no systematic study or evaluation of the CR mechanism at CONICET and its practical functioning. For it to be truly effective, regulatory provisions and implementation alone are insufficient. There is a need for mechanisms that ensure effective execution, as well as processes for identifying potential CRs and critically assessing their effectiveness.

### 2.9 Extraordinary/honorary professors at universities

The figure of EP appears in the statutes of many, if not all, universities. It is designed for individuals who, for various reasons, do not belong to the regular categories of ordinary professors (typically, plenary full professor, full professor, associate professor, and assistant professor; see details in Sections 3–6 in Supplementary material), but whose contributions to the university and academic community are considered valuable. The specific categories vary by university, but they commonly include consulting professors, emeriti professors, honorary professors, and visiting professors. This mechanism allows ordinary professors to continue their scientific and academic activities as consulting or emeritus professors after reaching retirement age. It also enables national and international figures of outstanding intellectual and artistic merit to be recognized by the university as honorary professors and facilitates collaboration between professors from other institutions, allowing them to temporarily engage in academic and scientific work as visiting professors. The regulations governing the appointment of EPs also vary by university. For example, Sections 3–5 in Supplementary material outline the relevant information in the statutes of three national universities: the University of Buenos Aires (UBA; Section 3 in the Supplementary Material), the National University of the South^11^ (UNS; Section 4 in Supplementary material), and the National University of Córdoba (UNC; Section 5 in Supplementary material).

Unlike CONICET, as far as we know, no category of EP in universities is explicitly designed to integrate Argentine researchers abroad who regularly collaborate with the university in academic and research activities. The categories of consulting and emeritus professors are typically reserved for those who have been regular professors. The honorary professor category is aimed at distinguished foreign and national figures, but it is an honorary distinction rather than an active affiliation. The visiting professor category does not distinguish between national and foreign individuals, but it is a temporary designation and does not aim to integrate visiting professors into the university. Recently, the National University of La Plata (UNLP) incorporated the CR figure into its policy (see details in Section 6 in Supplementary material), but these researchers hold temporary status, like visiting professors and therefore they constitute a vinculation, not an integration mechanism.

The notion of university affiliation for external members beyond the regular faculty is also rooted in the spirit of the 1918 movement referred to as the University Reform ^12^ (Del Mazo, 1941; Portantiero, 1978; Careño, 2018), one of whose principles is that of “free faculty” (*cátedra libre*) (see Sections 3, Art. 62, and 5, Art 62, in Supplementary material). This principle upholds the right of any qualified individual—whether intellectuals, scientists, or artists—to engage in teaching on an honorary basis provided the corresponding authorization. While the interpretation presented in these notes is broader and more flexible than the traditional definition, it is adapted to contemporary times and current needs. It encompasses academic activity in its entirety, possibly with a particular emphasis on research, technological development, and human resource training—going beyond the conventional understanding of teaching as simply giving lectures. Nonetheless, the fundamental principle of fostering a plurality of ideas remains.

### 2.10 The junior KP diaspora

The existing categories of CRs at CONICET and EPs in universities are designed for established researchers.

A forward-thinking integration policy must expand these categories to include junior researchers who are in the process of becoming established. Beyond representing the future senior KP diaspora, their perspective, particularly on emerging fields would critically complement that of the more senior population. Furthermore, it is important to maintain continuous institutional contact with KPs in training—doctoral candidates and postdoctoral researchers abroad. This is the group with the highest likelihood of returning to their CO to begin their professional careers there should the opportunity arise.

## 3 A policy framework

The following policy framework is tailored to the Argentine institutional context, primarily focusing on CONICET and universities. While grounded in the Argentine experience, it provides a scalable roadmap for a broad spectrum of STI institutions, and governmental and non-governmental organizations interested in policy-making to address the effects of brain drain through KP integration.

The list of specific recommendations provided here is extensive and detailed by design with the intention that they serve as a conceptual policy blueprint and facilitate the adaptation of the proposed framework to diverse national realities and varying scientific ecosystems

While we use the traditional term “corresponding” for historical clarity, we recognize that this terminology may be considered outdated in the evolving landscape of the KP diaspora policy.

### 3.1 Corresponding Researchers (CRs)—CONICET (“senior”)

These recommendations are based on the current (CONICET, 2015) and previous (CONICET, 1987, 2006) regulations and informed by the recent call for CRs (CONICET, 2022b,a) (see Supplementary material, Sections 1 and 2).

#### 3.1.1 Eligibility criteria

1. This category is intended for established researchers.
2. The minimum requirements should match those for independent (principal and senior) researchers in the regular career track.
3. In other national systems, this typically corresponds to the second or third career rank (e.g., Associate Professor with tenure in the U.S. system).

#### 3.1.2 Admission mechanism

1. Maintain the system of permanent admission (as in the 2015 policy).
2. Conduct periodic evaluations of this mechanism based on the number of yearly applications.

#### 3.1.3 Expansion of the list of proponents in the 2015 regulation

1. Inclusion of members of the above-mentioned committee.
2. Inclusion of chairs of the RCAE (Networks of Argentine Scientists Abroad).

#### 3.1.4 Evaluation and monitoring committee for CRs

1. Consisting of regular and corresponding researchers (CR).
2. Directly dependent on the CONICET Directorate.
3. Responsible for evaluating candidates for CR admission.
4. Facilitates the integration of CRs into respective institutes.
5. Monitors CR activities and evaluates their contributions.
6. Develops and implements mechanisms to identify potential CRs.

#### 3.1.5 Affiliations with institutions in Argentina

1. Adopt the requirement that CRs must be affiliated with at least one institution in Argentina proposed in the 2022 call.
2. Promote affiliations with research and academic institutions in disadvantaged or less central regions.

#### 3.1.6 Research areas

1. CRs must list their specific areas of research, development, or broader professional focus.
2. These areas should not be confined to the traditional “major knowledge areas” (CONICET) to allow for the inclusion of emerging fields and interdisciplinary collaborations.
3. A periodic and systematic review and update should be conducted to incorporate the continuously evolving fields, ensuring relevance to contemporary research landscapes. The current list of “major knowledge areas” is outdated and does not include new fields that have emerged since its creation—particularly interdisciplinary domains.

#### 3.1.7 Paid visits to Argentina

1. Implement a program of remunerated visits as a mechanism to enhance integration. Through this program, CRs will actively contribute to the local scientific community through.

(a) Teaching.
(b) Scientific and professional talks.
(c) Scientific collaborations.

2. Require that CRs should not only engage with their institutions of affiliation, but also extend their participation to other universities and research centers located in regions where scholars and students have less access to external visitors.

### 3.2 Junior Corresponding Researchers (JCRs)—CONICET

1. Create this category for researchers abroad who are in the process of becoming established researchers.
2. The minimum eligibility requirements should match those for adjunct researchers in the regular CONICET research career track.
3. In other national systems, this corresponds to the first rank of the research career (e.g., Assistant Professor or Associate Professor without tenure in the U.S. system).
4. Incorporate JCRs into the Evaluation and Monitoring Committee.
5. Extend all relevant recommendations made for the CR category to the JCR category as necessary and appropriate.

### 3.3 Corresponding Researchers in Training (TCRs)—CONICET

1. Create this category for doctoral candidates and postdoctoral researchers completing their training in foreign universities (and residing abroad).
2. Design international collaboration mechanisms aimed at reintegration of the TCRs to the local systems once their training abroad is completed.
3. Develop reintegration (return) mechanisms for TCRs upon completing their training abroad.
4. Design mechanisms to promote the continuation of the collaborations started abroad upon return of the TCRs.
5. Extend all relevant recommendations made for the CR and JCR categories to the TCR category as necessary and appropriate.

### 3.4 Extraordinary/honorary professors/researchers (“abroad”) at universities

These recommendations extend the figure of CR to universities. They are informed by the statutes and regulations of four Argentine universities (see Sections 3–6 in Supplementary material): University of Buenos Aires (UBA), National University of Cordoba (UNC), National University of the South (UNS, Bahía Blanca) and National University of La Plata (UNLP).

1. Create a new category or regulate an existing category following the general guidelines and objectives of the CONICET's CR program (described above).
2. Establish Evaluation, Monitoring, and Identification Committeed, following the general guidelines described earlier for CRs at CONICET.
3. Implement a paid visit mechanism similar to the one outlined for the CONICET CRs.
4. Extend this category to junior and “in training” researchers following the guidelines described earlier for the corresponding CONICET JCR and TCR categories.

### 3.5 Similar categories in other research and technology institutions

In the case of Argentina, these institutions primarily include

1. INTA (National Institute of Agricultural Technology).
2. INTI (National Institute of Industrial Technology).
3. CNEA (National Atomic Energy Commission).
4. CONAE (National Space Activities Commission).

### 3.6 Interdisciplinary and inter-institutional evaluation and update committees

#### 3.6.1 Composition

1. Regular researchers from CONICET and other research and development institutions (INTA, INTI, CNEA).
2. CRs from CONICET.
3. KPs in similar categories created in other research and development institutions and universities.
4. Representatives from the National Interuniversity Council (CIN) and the Council of Private University Rectors (CRUP).
5. Representatives of the RCAE.
6. Representatives from science policy bodies (formerly MinCyT).
7. Representatives from the foreign service (*Canciller*í*a*).

#### 3.6.2 Responsibilities

1. Continuous evaluation of the integration mechanisms within the framework of public policy.
2. Evaluation of joint research output between CRs and regular CONICET and university members.
3. Updating integration mechanisms in the context of public policy as needed.
4. Examining and resolving issues related to intellectual property and patents associated with the integration policies and mechanisms.
5. Assessing the possibility of incorporating industry-based KPs as CRs or equivalent in universities.
6. Addressing potential issues that may arise.
7. Produce a yearly evaluation report.

#### 3.6.3 Intellectual property

1. Evaluate intellectual property issues.
2. Design a flexible intellectual property agreement policy to apply when necessary.

### 3.7 Development of research projects

These research projects should aim to achieve the following goals

1. Assess the institutional, social, and economic impact of proposed public policies and KP diaspora integration mechanisms.
2. Validate the core hypotheses underpinning the presented recommendations.
3. Analyze alternative hypotheses and theoretical frameworks.
4. Explore additional mechanisms of KP diaspora integration, as required by shifting contexts.
5. Generate evidence-based recommendations to further refine the policy framework.
6. Evaluate the economic impact and quantify the sustainability and value-added of the proposed policies.

## 4 Conclusion

The ideas and policy framework developed in this paper are situated within the field of STI diplomacy (Gual Soler, 2020, 2021; Lima-Toivanen et al., 2025; Echeverría-King et al., 2022), with a particular emphasis on developing international collaborations and addressing the brain drain phenomenon by focusing on tight international collaborations between members of the KP diaspora and their colleagues in their CO. We operationalized the concept of KP diaspora integration (Rotstein and Galvis, 2023) through a structural membership model enabling the diaspora of KPs to maintain affiliations with domestic STI institutions via courtesy appointments. This is supported by a comprehensive policy framework of specific steps or recommendations to facilitate, and even drive, the involvement of the KP diaspora in the STI activities of their CO and the development of international collaborations between domestic and diaspora KPs. This proposition constitutes a qualitative shift from the traditional approaches focused primarily on vinculation and repatriation and sometimes limited to them. While we argue integration is a strategy distinct from these traditional models, we acknowledge that no single approach is “one-size-fits-all.”

The proposed policy framework is conceived as a comprehensive blueprint. We utilized Argentina as a case study for the several reasons elaborated in the Introduction and documented along the paper.

While grounded in the Argentine context, the underlying principles and policy blueprint possess a broader relevance, applying *mutatis mutandis* to other developing nations facing similar systemic challenges Organización Internacional para las Migraciones (Autor Institucional), 2007(Echeverría-King et al., 2022; Echeverría-King et al., 2025; Pombo et al., 2025; Piñeros Ayala et al., 2025; Coelho Ferreira et al., 2025; Hernández Mondragón et al., 2025).

Institutions adopting this framework, or parts of it, are expected to tailor its components to their specific realities and institutional, local, regional and national needs.

This process requires a feasibility analysis at the institutional level, taking into account local culture and existing organizational structures. Implementation costs are expected to be negligible compared to standard STI expenses, as the model involves no regular salaries; the only recurring costs are travel expenses for on-site visits, should the institution choose to implement that specific policy component. Furthermore, since the designation of honorary faculty is typically already present in university statutes, implementation would only require the adaptation of internal regulations rather than fundamental changes to constitutional documents. Examples are presented in Sections 4 (UNS) and 6 (UNLP) in Supplementary material. We note that in the case of UNS, the modifications to the regulations were done after the circulation of a working paper preceding this one (Rotstein, 2025a) and after personal conversations between the author and he UNS leadership.

From this point of view, the implementation of the proposed framework is likely to encounter minimal institutional resistance. On the other hand, the implementation of the specific policies may not be free from conflict. While KP diaspora members holding a courtesy appointment lack formal voting rights, they might have a voice, even it is informally, on certain aspects of institutional policy or research directions without have to live with the consequences of these decisiones. We cannot rule out the possibility of this creating resentment among the domestic KP population. This should be address during the implementation policy and continuously evaluated.

A critical component of the proposed framework is the requirement for reflexive evaluation, ensuring that both the policies and their tangible results are systematically assessed. While this is framed at the high-level policy stage, institutions adopting the framework may choose to conduct similar internal evaluations to ensure the model remains aligned with their evolving needs and goals. Complementing this, the framework emphasizes the development of research projects aimed at assessing the STI and socio-economic impacts of the policy, validating underlying hypotheses, and exploring alternative integration mechanisms to generate refined recommendations and necessary adjustments.

The proposed framework implicitly change the organizational architecture governing the link between the KP diaspora and the STI institutions of their CO by shifting from rigid and vertical structures to horizontal, decentralized networks, having multiple bidirectional nodes (e.g., research institutes, universities, STI government offices, embassies/consulates) interacting at multiple levels (e.g., research groups, university departments, university administration, STI government offices) as opposed to classical top-down (e.g., STI government office to the KP diaspora) o hybrid between top-down / bottom-up approaches (Rotstein, 2023). Research is needed to examine these architectures and determine the optimal ones. The characteristics of knowledge diaspora networks have been examined by other authors (Lacerda et al., 2023; Granés et al., 1996; Chaparro et al., 2006, 2004; Meyer, 2001, 2011; Kuznetsov, 2006; Fink and Miguelez, 2017; Agrawal, 2017; Meyer and Wattiaux, 2006; Epstein and Heizler, 2016).

The ideas and concepts developed in this paper are informed by a synthesis of existing literature and public policy documents, further motivated by the author's professional trajectory as an active member of the Argentine KP diaspora. This perspective is shaped by extensive engagement with the Argentine STI system—spanning scientific research, teaching, mentoring, scientific meetings organization, and science diplomacy—specifically through roles as a Corresponding Researcher (CR) at CONICET and within the leadership of the Networks of Argentine Scientists Abroad (RCAE). While these personal insights provide a nuanced vantage point, the framework is developed through a systemic lens and grounded in an analysis and documentation that transcends individual experience.

## Data Availability

The original contributions presented in the study are included in the article/Supplementary material, further inquiries can be directed to the corresponding author.

## Footnotes

1 rcae.info (Accessed April 10, 2026).

2 One example is the Milstein Fellowship https://www.argentina.gob.ar/ciencia/financiamiento/subsidios-milstein (Accessed November 05, 2025), which “… aim to strengthen the connection between Argentine researchers residing abroad and the local scientific and technological community. These grants fund stays of up to 3 months, enabling researchers to engage in academic collaborations, participate in scientific activities, and contribute to knowledge exchange within Argentina.”

3 “[They] Returned to Argentina in 1984, but they were not happy with the work environment in the country, and then accepted an invitation to return to Unicamp (in Brazil) in 1988.” “Now my roots are here. I have a son, a son in law, and four Brazilian grandchildren.” https://revistapesquisa.fapesp.br/es/a-50-anos-de-aquella-noche/ (Accessed May 12, 2025).

4 This is a shared zone between repatriation and circulation.

5 https://www.argentina.gob.ar/ciencia/raices/premios (Accessed May 11, 2025).

6 https://www.argentina.gob.ar/ciencia/raices/subsidio-milstein.

7 It is often believed that the goal of the RAICES Program is limited to the repatriation of scientists, although the same papers describe other activities of the RAICES Program that go beyond the repatriation of scientists [e.g., (Trotman, 2022); see also (Pombo et al., 2025; Vera and López, 2023)].

8 ANACITEC (Asociación Argentina Norteamericana para la Ciencia, la Tecnología y la Cultura), ECODAR (Encuentro Cooperación Diáspora-Argentina), PROCITEXT (National Program for Engagement with Argentine Scientists and Technologists Abroad), CAPICCyTE (Comité de Asesores de Programas de Cooperación Científica y Tecnológica en el exterior), CEGA (Centro de Estudiantes y Graduados de la Argentina), APUA Asociación de Profesionales Argentinos en Alemania), AESFA (Asociación para los Intercambios Científicos Franco-Argentinos; Association pour les Échanges Scientifiques Franco-Argentins), APARU (Asociación de Profesionales Argentinos en el Reino Unido).

9 rcae.info (Accessed April 10, 2026).

10 https://convocatorias.conicet.gov.ar/carrera-del-investigador/correspondiente/ (Accessed March 20, 2025).

11 Universidad Nacional del Sur, Bahía Blanca.

12 The Reforma Universitaria (1918) was an important university political movement in Argentina, led by students, who demanded modernization of the curricula and participation in the university governance among others.
