# Supplementary material for: Integration between the knowledge diaspora and their country of origin through institutional affiliations: a case study for policy development
Source: Front Res Metr Anal. 2026 Jul 3;11:1852906. doi: 10.3389/frma.2026.1852906 (PMC13375875; doi:10.3389/frma.2026.1852906)
Supplement: Supplementary file 1 [file Data_Sheet_1.pdf]

Supplementary Material

1 THE NOTION OF INTEGRATION IS PRESENT IN THE CONICET POLICY THAT ESTABLISHED THE CORRESPONDING RESEARCHER (CR) CATEGORY IN 1987 AND PERSISTS IN SUBSEQUENT POLICIES

The Corresponding Researcher (CR) category at CONICET (National Council of Scientific and Technological Research) was established in 1987 under the name "Corresponding Member of the Scientific and Technological Researcher Career" (CONICET, 1987). The present term "Corresponding Researcher" (*Investigador Correspondiente*) of CONICET was first used in the 2006 policy (CONICET, 2006) and remained in the 2015 amendment (CONICET, 2015). Here, we refer to these policies as the 1987, 2006, and 2015 policies. While the 2006 policy states that the CR category was "created," in practice, it was a modification of the 1987 policy with a name change. In 2022, a call for applications (CONICET, 2022b,a) was made based on the 2015 policy.

Specifically, the notion of "integration" can be traced back to the 1987 policy (in the "whereas clauses" and Articles 3 and 4) and remains implicitly present in the 2006 and 2015 policies. Below, we present the translation to the most relevant sections.

Whereas clauses:

(i) That the historical development of science and technology in our country has led to the formation of significant human resources, and that a considerable number of them are practically permanently settled abroad.

(ii) That the democratic government has defined the "Argentine scientific community" as the group of all Argentines dedicated to research in various disciplines, whether in the country or abroad.

(iii) That within the two aforementioned groups, there is a considerable number of researchers willing to collaborate in strengthening the scientific and technological system in our country without changing their current workplaces.

(iv) That it is considered advisable to strengthen ties between these scientists and those residing in the country, particularly with this Council.

(v) That numerous researchers have expressed interest in creating mechanisms that facilitate and standardize these connections to promote exchanges of people, training of human resources, and cooperation initiatives.

Articles:

3. Evaluations for admissions, projects, or other actions related to the regime established by this resolution will be conditioned by their impact on integration with the Argentine scientific community, the training of human resources, scientific equipment, and international relations.
4. Researchers who are Argentine nationals, habitually residing abroad, and who wish to maintain a regular, not sporadic, relationship with the Argentine scientific community, the country's universities, and CONICET may apply for admission as Corresponding Members.

2 EVOLUTION OF THE POLICIES FOR CONICET CORRESPONDING RESEARCHERS (CR)

Scientific and Technological Researcher Career (CONICET). The “regular” track is governed by a Statute (Law, 1973), a Ranking System (Decree, 1976), and policies and other regulations (laws, decrees). The five “categories” in this career, as defined in the statute, are Assistant Researcher, Associate Researcher, Independent Researcher, Principal Researcher, and Senior Researcher, listed in ascending order of requirements.

Eligibility requirements for CRs. In the 1987 policy, it was established that the minimum eligibility requirements are the same as those for the three highest regular categories (Independent Researcher, Principal Researcher, and Senior Researcher), with mention of this category. The subsequent policies (2006, 2015) do not explicitly define minimum eligibility requirements.

Duration. In the 1987 policy, it was established that the CR designation lasted six years. The subsequent policies (2006, 2015) do not set expiration limits for the CR designation.

Visits to the country (Argentina). The 1987 policy included provisions for CRs to visit the country, sponsored by CONICET, with paid expenses (airfare and a stipend). It also established a minimum requirement of one such visit every six years. This requirement is absent in the later policies (2006, 2015).

Reports. With some variations, all three policies (1987, 2006, 2015) establish the requirement for submitting a scientific activity report. The current policy (2015) specifically requires a biennial report detailing the specific activities carried out within the framework of the CR recognition.

Admission. In the 1987 regulation, admission could occur either by:

- The applicant’s request.
- A special invitation facilitated through the CONICET Board of Directors.

The 2006 and 2015 policies maintain the first option and expand the second by explicitly stating that admission can also be proposed by:

- The President of CONICET.
- A member of the CONICET Board of Directors.
- Directors of Executive Units (e.g., research units)
- Rectors (Presidents) of public or private universities.
- Scientific associations or societies.

Approval of Admission: Regular provision. The 1987 policy does not establish specific criteria, implying that approval is subject to the usual rules of the CONICET Board of Directors. The 2006 policy states that approval by the Board of Directors must be unanimous. The 2015 policy indicates that the proposal must be approved by the Board of Directors but does not specify a requirement for unanimity.

Approval of Admission: Special provision. In the 2022 call for applications, it was established that applications would be evaluated by a special committee according to the procedures set forth in the policies governing the regular CONICET evaluation system. Across the 1987, 2006, and 2015 policies, it is implicitly understood that evaluations follow CONICET’s standard mechanisms.

Disciplines. The 2006 and 2015 policies establish the requirement to select one of the major areas of knowledge (CONICET's broad disciplinary areas). The 1987 regulation does not mention this requirement.

Commitments. The 2006 and 2015 regulations outline a set of commitments undertaken by CRs:

- Promoting national and international cooperation.
- Encouraging the organization of colloquia, seminars, conferences, and other research and development activities with specialists from both Argentina and abroad.
- Participating in the evaluation of scientific and technological activities upon request by CONICET.

Formal Connection with Institutions in Argentina. A notable update in the 2022 call for applications is the requirement that CRs maintain a formal affiliation with an institution in Argentina. It is recommended that this institution be an executive unit (e.g., a research institute) of CONICET. While it could be thought to be implicit in the current (2015) and previous (1987, 2006) policies, it is not formally required.

3 EXTRAORDINARY PROFESSORS: UNIVERSITY OF BUENOS AIRES (UBA), ARGENTINA

The statute of UBA establishes [1]

Article 34. Professors at the University of Buenos Aires fall into the following categories:

1. Regular Professors:
   (a) Full Professors, Associate Professors, and Assistant Professors
   (b) Adjunct Professors
2. Consulting Professors
3. Contracted and Visiting Professors
4. Emeritus and Honorary Professors

Additionally, *ad honorem* (honorary) educators collaborate in teaching, including authorized and independent ("free") faculty (educators).

Article 52. Consulting Professors collaborate in the delivery of special courses for students and graduates and continue their research activities, all with the approval of the Board of Directors. The provisions of Article 44 regarding the renewal and expiration of their designation apply to Consulting Professors.

Article 53. Consulting Professors may be part of any governance and advisory bodies of the University.

Article 54. Contracted and Invited Professors are faculty members or researchers of different categories whom each Faculty may invite or hire, with the stipulated remuneration and for the agreed-upon duration. Contracted or invited Professors and researchers will belong to a category suitable for the tasks deemed necessary by the respective Faculty. To proceed with a contract or invitation, the Faculty must secure

[1] University of Buenos Aires, University Statute, organized Text
(i) https://servicios.infoleg.gob.ar/infolegInternet/verNorma.do;jsessionid=90799712B86DCBBE3D025A9D285A3F09?id=318558 (Accessed: 2025-05-13)
(ii) https://servicios.infoleg.gob.ar/infolegInternet/anexos/315000-319999/318558/norma.htm (Accessed: 2025-05-13)
(iii) http://www.fcen.uba.ar/prensa/noticias/documentos/estatutouba.html (Accessed: 2025-05-13).

approval from two-thirds of the members of its (Faculty) Board of Directors. Additionally, authorization from the (University) Higher Council is required upon a justified request by the Faculty.

Article 55. Extraordinary Professors are appointed by the University's Higher Council based on a well-founded proposal from one of its members or a Faculty, recognizing exceptional merit. They are classified into two categories: Emeritus and Honorary.

Article 56. Emeriti Professors are full or tenured professors who have reached the age of sixty-five and who, due to demonstrating extraordinary capabilities in both teaching and research, are proposed for this category by the Board of Directors of the respective Faculty, requiring a unanimous vote from its members.

Article 57. Emeriti Professors may continue their research, collaborate in teaching students or graduates, and be part of any governance bodies within the University. If Emeriti Professors wish to continue their research, Faculties must take necessary measures to facilitate their work.

Article 58. Honorary Professors are distinguished individuals in the intellectual or artistic field, whether from Argentina or abroad, whom the University specially honors with this designation.

Article 59. Authorized Faculty assist professors in university tasks. The title of Authorized Faculty is granted by the Higher Council to those who have completed their academic training in accordance with each Faculty's regulations.

Article 61. Authorized faculty retain their status until the age of sixty-five, unless the Board of Directors requests their termination due to non-compliance with current regulations.

Article 61. The activity of authorized faculty is compatible with teaching courses and leading research projects or practical work.

Article 62. Independent (free) faculty are individuals authorized by a Faculty's Board of Directors to teach new or parallel courses alongside existing ones. Authorization is granted upon request by interested parties or faculty members, under the conditions and for the duration established by the Boards of Directors of the Faculties.

The policies for the Extraordinary Professor (PE) categories can be found in the UBA Code, Book I, Title 10, under the following chapters: Chapter B: Consulting Professor [2], Chapter C: Emeritus Professor [3], Chapter D: Invited and Contracted Professors [4], Chapter E: Honorary Professor [5].

[2] `https://codigo.rec.uba.ar/codigo_uba/libro-i-normas-generales-de-la-universidad-de-buenos-aires-1/titulo-10-profesores/capitulo-b-profesores-consultos/` (Accessed: 2025-05-13)

[3] `https://codigo.rec.uba.ar/codigo_uba/libro-i-normas-generales-de-la-universidad-de-buenos-aires-1/titulo-10-profesores/capitulo-c-profesores-emeritos/` (Accessed: 2025-05-13)

[4] `https://codigo.rec.uba.ar/codigo_uba/libro-i-normas-generales-de-la-universidad-de-buenos-aires-1/titulo-10-profesores/capitulo-d-profesores-invitados-y-contratados/` (Accessed: 2025-05-13)

[5] `Honoraryprofessorhttps://codigo.rec.uba.ar/codigo_uba/libro-i-normas-generales-de-la-universidad-de-buenos-aires` `titulo-10-profesores/capitulo-e-profesor-honorario/` (Accessed: 2025-05-13)

4 EXTRAORDINARY PROFESSORS: UNIVERSIDAD NACIONAL DEL SUR (NATIONAL UNIVERSITY OF THE SOUTH; UNS), BAHÍA BLANCA, ARGENTINA

The Statute of UNS) establishes [6]

Article 15. Extraordinary Professors belong to one of the following categories:

a) Honorary Professor
b) Emeritus Professor
c) Consulting Professor
d) Visiting Professor

The requirements for each category, as well as the appointment period, obligations, and privileges, will be regulated by the University's Higher Council (*Consejo Superior Universitario*).

Based on recommendations from the University Assembly [7], in Chapter I of the policies for the appointment of extraordinary professors [8], the University's Higher Council (CSU) of UNS established

Article 2. The Honorary Professor category is granted to distinguished national or international figures whose outstanding merits earn them this special recognition from the University.

Article 3. The categories of Consulting Professor and Emeritus Professor are conferred upon professors who, after a long and distinguished career at UNS, qualify for the highest retirement benefits and are honored by the University in recognition of their achievements.

Article 4. A Consulting Professor is an individual of extraordinary merit in their scientific, technical, or humanistic field, whose contribution to university life and the training of human resources has gained broad recognition from peers and students. Their counsel and participation are considered academically valuable for the sustained development of an Area or Department.

Article 5. An Emeritus Professor is a scholar of undeniable academic stature, who has achieved broad national and international recognition and thus serves as a model of the academic and scientific excellence to which the Institution aspires.

Article 6. The Visiting Professor category applies to professors from other national or international universities, or to distinguished figures in Science, Technology, or the Arts, who are invited to undertake temporary academic activities at UNS.

This policy contains additional information about the categories of Honorary Professors (Chapter II), Consulting and Emeriti Professors (Chapter III), and Visiting Professors (Chapter IV). The procedures and responsibilities in the appointment process for extraordinary professors are established in two subsequent resolutions issued by the Higher University Council (CSU) of UNS.

[6] Statue of UNS, organized text, 2005 `https://servicios.uns.edu.ar/institucion/conc_nd/docs/estatuto_uns.pdf` (Accessed: 2025-05-14)

[7] UNS-AU-5/88. General Guidelines for the Appointment of Extraordinary Professors `https://apps.uns.edu.ar/digesto-consultas/bof/digesto_busqueda_resu.php` (Accessed: 2025-05-13)

[8] UNS-CSU-118/93. Policies for the Appointment of Extraordinary Professors `https://apps.uns.edu.ar/digesto-consultas/bof/digesto_busqueda_resu.php` (Accessed: 2025-05-13)

On December 18, 2025, after the publication of a preliminary preprint version of this article, the University's Higher Council (CSU) of UNS modified the above-mentioned regulation regarding Honorary Professors as follows [9]

Article 2. The category of Honorary Professor is reserved for distinguished national or international figures of exceptional merit who are not members of the permanent faculty of the *Universidad Nacional del Sur*, and whose professional trajectory and contributions may significantly enhance the holistic education of the University's undergraduate and graduate students, as well as the expansion of the institution's global network.

Article 7. Designation as Professor Emerita/Emeritus, "Consulta/Consulto", or Honorary Professor constitutes an *ad honorem* academic distinction, entailing no obligation to reside within the sphere of influence of the *Universidad Nacional del Sur*, nor does it, in and of itself, confer any right to remuneration. In the case of Professors Emeriti or Consultas/Consultos, this distinction shall be for life and shall not require the performance of specific duties within the institution.

Article 11. Honorary Professors may make use of the University's facilities, libraries, and laboratories, as well as propose and teach undergraduate and graduate courses; co-direct and participate in research projects; co-direct and participate in extension projects; and undertake academic, productive, or social activities that contribute to institutional development and student education—always in coordination and collaboration with faculty members and academic units of the *Universidad Nacional del Sur*.

Honorary Professors must maintain an active affiliation with the University, participating in activities consistent with the institution's mission. Either party may, at its sole discretion, terminate this affiliation at any time through an administrative process similar to that of the initial appointment (ARTICLE 14). The department initiating the appointment process shall be responsible for maintaining an up-to-date record—updated at appropriate intervals—of the activities undertaken and the grounds justifying the continuation of the appointment.

This appointment is compatible with other affiliations or contracts with the University, provided that they do not give rise to a conflict of interest or an overlap of duties.

Article 14. The designation of an Honorary Professor shall be made upon the proposal of an Academic Department of the UNS. The submission must include, at a minimum, an up-to-date curriculum vitae of the candidate, information regarding their affiliation with the institution, a description of their impact within the academic, productive, and/or social spheres, and an academic justification for their selection—highlighting their potential contribution both to the education of undergraduate and graduate students and to the development of the University's core functions, as well as their role in articulating efforts to expand and strengthen the institution's global engagement network.

5 EXTRAORDINARY PROFESSORS: NATIONAL UNIVERSITY OF CÓRDOBA (UNC), ARGENTINA

The Statute of the National University of Córdoba (UNC) establishes [10]

[9] UNS-CSU-914/25. Modifies Honorary Professor Regulations `https://apps.uns.edu.ar/digesto-consultas/bof/digesto_busqueda_resu.php` (Accessed: 2026-04-03)

[10] `https://www.unc.edu.ar/sobre-la-unc/estatuto` (Accessed: 2025-11-14)

Article 62. Professors at the National University of Córdoba (UNC) fall into the following categories:

1) Regular Professors
   a) Full Professors
   b) Tenured Professors
   c) Associate Professors
2) Assistant Professors
3) Auxiliary Profesores (teaching assistants)
4) Consulting Professors and Emeriti Professors
5) Honorary Professors
6) Contracting Professors and Visiting Professors

Additionally, Authorized Faculty and Independent Faculty (Free Faculty) collaborate in teaching but do so without remuneration.

Article 71. The appointment of a Consulting Professor is proposed by the Faculty's Board of Directors or the Rector (President) to the Higher Council. To receive this distinction, the candidate's academic trajectory must be relevant, and their appointment requires an absolute majority from both the Board of Directors and the Higher Council.

Article 72. The Consulting Professor collaborates in the delivery of special courses for students and graduates or continues research activities, all with the approval of the Board of Directors. The provisions of Article 67 regarding the renewal and expiration of the appointment apply to Consulting Professors.

Article 73. An Emeritus Professor is a Full Professor[11] who has obtained retirement benefits and who, due to demonstrating extraordinary qualities in teaching, research, and/or university outreach, is proposed for this category by the Faculty's Board of Directors with a two-thirds majority vote or by the Rector and is appointed with an absolute majority by the Higher Council. The conditions established in Article 72 also apply to Emeritus Professors.

Article 74. Honorary Professors are eminent figures in the intellectual or artistic fields, either from Argentina or abroad, whom the University honors with this designation in accordance with current regulations.

Article 75. Contracted and Visiting Professors are faculty members or researchers of various ranks whom each Faculty may invite or hire with the stipulated remuneration and for an agreed-upon duration. Contracted or invited Professors and researchers will belong to a category suitable for the necessary tasks determined by the respective Faculty. To proceed with a contract or invitation, the Faculty must secure approval from two-thirds of its Board of Directors' members. Additionally, authorization from the Higher Council is required upon a justified request by the Faculty.

[11] There is no distinction in English between "profesor titular" y "profesor titular plenario"

The regulations for the Extraordinary Professor (PE) categories can be found in the Teaching Policies [12] for Consulting and Emeritus Professors [13] and Honorary Professors [14]. However, no information was found regarding the regulations for Visiting and Contracted Professors.

6 CORRESPONDING RESEARCHER: UNIVERSIDAD NACIONAL DE LA PLATA (UNLP), ARGENTINA

The 2024 Ordinance 284 [15] "Research Units: Creation, Recognition, Typification, and Evaluation," updated the 2016 Ordinance [16] and introduced the Corresponding Researcher category.

Article 5. The permanent members in the Research Units are faculty researchers, researchers with exclusive workplace affiliation within the unit, graduate students[17], scholarship holders, Extraordinary Professors (Emeriti and Consulting), Non-teaching staff, members of Technical or Professional Support Careers (as defined by a specific agreement). The temporary members are the corresponding researchers and visitors (both for a defined period and with exclusive responsibility for fulfilling authorized tasks within the unit)

Article 6. . It is recognized as a corresponding researcher of a Research Unit, a person who performs temporary research or supervision tasks (direction or co-direction) for another trained or trainee researcher, fellow, thesis student, or other recognized categories in that Research Unit. To be recognized as such, a plan of tasks and the duration of the task to be performed must be submitted to the Faculty Board of Directors. It must be endorsed by a Researcher of the Research Unit and approved by the Board of Directors. Any extension period or condition must be approved by the Board of Directors itself. Trained researchers (equivalent to categories DI1, DI2, or DI3 of SICADI) from any recognized national or international university or research organization may apply for this status. Being a corresponding researcher does not imply an employment relationship, nor does it entitle them to elect peer representatives or to join any Management[18] body of the Research Unit that accepts them in this capacity. Corresponding researchers must incorporate this affiliation into their scientific production resulting from the tasks recognized in the work plan endorsed for this appointment.

7 DEFINITIONS

Knowledge Professionals. It refers to individuals whose primary contribution to the economy and society is the creation, application and distribution of theoretical and practical knowledge, and the management of information and specialized expertise.

Brain drain. It refers to the significant loss of educated and highly skilled citizens due to emigration, most dramatically from developing countries to industrialized economies.

[12] https://www.unc.edu.ar/academicas/normativa-docentes (Accessed: 2025-05-14)
[13] Accessed: 2025-05-14
[14] Accessed: 2025-05-14
[15] https://idihcs.fahce.unlp.edu.ar/wp-content/uploads/2024/08/Ordenanza-284-aprobada-2024.pdf (Accessed: 2025-11-20)
[16] https://www.exactas.unlp.edu.ar/uploads/docs/ordenanza_284_16.pdf Accedido: 2025-11-20)
[17] Working on their dissertation/thesis
[18] Governance

Brain gain. It refers to the economic and social benefit a country experiences when it receives an influx of highly skilled, educated, and professional individuals from other nations.

Brain vinculation (linkage). It refers to a policy framework adopted by a country focused on establishing and maintaining formal ties with its KP diaspora. It leverages the KP diaspora as a non-local asset and takes advantage of the potential of collaboration with the countries of residence of the diaspora members. Existing mechanisms include fostering diaspora networks that allow their members to contribute to their country of origin without returning permanently by organizing and/or participating in activities such as virtual workshops, virtual teaching, and research collaborations.

Brain circulation. It refers to the policies aimed at sending KPs abroad for training and education purposes with the explicit commitment of returning to their country of origin to continue developing their careers with the expected positive consequences for the local economies. For example, KPs may move abroad to gain expertise on a certain topic or technology and then return to their country of origin with new skills. Existing mechanisms include training fellowships and sabbatical leaves.

Repatriation. it refers to the return of highly skilled knowledge professionals to their country of origin after a significant period of working or studying abroad.

Courtesy appointment. It is a non-permanent, typically unpaid, non-tenure-track academic position granted to someone already employed in another department, another university or elsewhere, recognizing their contributions (e.g., guest lecturing, advising, research collaboration) without forming an employer-employee relationship and without granting voting rights.

REFERENCES

CONICET (1987). Resolución 1636/87 (1987-10-15) del directorio del CONICET, Argentina, firmada por el entonces presidente, Dr. carlos abeledo

CONICET (2006). Resolución 2462/06 (2006-10-17) del directorio del CONICET, Argentina, firmada por el entonces presidente, Dr. eduardo h. charreau

CONICET (2015). Resolución 1351/15 (2015-04-21) del directorio del CONICET, Argentina, firmada por el entonces presidente, Dr. roberto salvarezza

CONICET (2022a). Resolución 774/23 (2023-05-11) del directorio del CONICET, Argentina, firmada por la entonces presidenta, Dra. ana maría franchi

CONICET (2022b). Resolución 887/22 (2022-07-18) del directorio del CONICET, Argentina, firmada por la entonces presidenta, Dra. ana maría franchi

Decree (1976). Decreto 1572/76, Carrera del Investigador Científico y Tecnológico y Carrera del Personal de Apoyo a la Investigación y Desarrollo. Escalafón. Aprobado: 1976-07-30. *https://www.argentina.gob.ar/normativa/nacional/decreto-1572-1976-32633/actualizacion (Accessed: 2025-05-12)*

Law (1973). Ley 20.464, Argentina. Estatuto de las carreras del investigador científico y tecnológico y del personal de apoyo a la investigación y desarrollo. Sancionada y promulgada: 1973-23-05. *https://www.argentina.gob.ar/normativa/nacional/ley-20464-60507/actualizacion (Accessed: 2025-05-12)*
